# Heparin-Loaded Alginate Hydrogels: Characterization and Molecular Mechanisms of Their Angiogenic and Anti-Microbial Potential

**DOI:** 10.3390/ma15196683

**Published:** 2022-09-26

**Authors:** Ayesha Nawaz, Sher Zaman Safi, Shomaila Sikandar, Rabia Zeeshan, Saima Zulfiqar, Nadia Mehmood, Hussah M. Alobaid, Fozia Rehman, Muhammad Imran, Muhammad Tariq, Abid Ali, Talha Bin Emran, Muhammad Yar

**Affiliations:** 1 Interdisciplinary Research Center in Biomedical Materials, COMSATS University Islamabad Lahore Campus, Lahore 54000, Pakistan; 2 Department of Biology, Lahore Garrison University, Lahore 54810, Pakistan; 3 Faculty of Medicine, Bioscience and Nursing, MAHSA University, Jenjarom 42610, Selangor, Malaysia; 4 Department of Zoology, College of Science, King Saud University, Riyadh 11362, Saudi Arabia; 5 Biochemistry Section, Institute of Chemical Sciences, University of Peshawar, Peshawar 25120, Pakistan; 6 Department of Medical Laboratory Technology, University College of Duba, University of Tabuk, Tabuk 71491, Saudi Arabia; 7 Department of Zoology, Abdul Wali Khan University, Mardan 23200, Pakistan; 8 Department of Pharmacy, BGC Trust University Bangladesh, Chittagong 4381, Bangladesh; 9 Department of Pharmacy, Faculty of Allied Health Sciences, Daffodil International University, Dhaka 1207, Bangladesh

**Keywords:** heparin, alginate, hydrogel, NIH3T3 fibroblasts, angiogenesis, antimicrobial, wound healing

## Abstract

**Background:** Chronic wounds continue to be a global concern that demands substantial resources from the healthcare system. The process of cutaneous wound healing is complex, involving inflammation, blood clotting, angiogenesis, migration and remodeling. In the present study, commercially available alginate wound dressings were loaded with heparin. The purpose of the study was to enhance the angiogenic potential of alginate wound dressings and analyze the antibacterial activity, biocompatibility and other relevant properties. We also aimed to conduct some molecular and gene expression studies to elaborate on the mechanisms through which heparin induces angiogenesis. **Methods:** The physical properties of the hydrogels were evaluated by Fourier transform infrared spectroscopy (FTIR). Swelling ability was measured by soaking hydrogels in the Phosphate buffer at 37 °C, and cell studies were conducted to evaluate the cytotoxicity and biocompatibility of hydrogels in NIH3T3 (fibroblasts). Real-time PCR was conducted to check the molecular mechanisms of heparin/alginate-induced angiogenesis. The physical properties of the hydrogels were evaluated by Fourier transform infrared spectroscopy (FTIR). **Results:** FTIR confirmed the formation of heparin-loaded alginate wound dressing and the compatibility of both heparin and alginate. Among all, 10 µg/mL concentration of heparin showed the best antibacterial activity against *E. coli*. The swelling was considerably increased up to 1500% within 1 h. Alamar Blue assay revealed no cytotoxic effect on NIH3T3. Heparin showed good anti-microbial properties and inhibited the growth of *E. coli* in zones with a diameter of 18 mm. The expression analysis suggested that heparin probably exerts its pro-angiogenetic effect through VEGF and cPGE. **Conclusions:** We report that heparin-loaded alginate dressings are not cytotoxic and offer increased angiogenic and anti-bacterial potential. The angiogenesis is apparently taken through the VEGF pathway.

## 1. Introduction

A wound is damaged skin or tissue caused by different reasons such as surgery, chemicals, trauma and extreme temperature. Wounds can also be due to other factors such as local blood supply disorders, cuts, burns and injuries. Injuries in tissue activate physiological responses which then initiate the process of wound healing [1]. After skin injury, the healing process starts with the formation of a clot, which is formed when a number of tissue factors activate the aggregation of platelets, which results in the release of chemokines and growth factors [2] Wound healing is basically the restoration of the damaged skin through a cascade of events including proliferation, differentiation, migration and apoptosis [3].

Wound management is still a challenge and a cost-effective wound dressing with angiogenic and antibacterial potential is yet to be developed [4,5]. There are a number of factors behind this, including ineffective clinical trials, poor research and a lack of critical skills and resources [6]. In wound management, most of the hurdles occur in the form of bacterial infections, skin allergies, poor nutrition, dehydration and psychosocial factors such as depression, insomnia and lack of self-care [7].

Nowadays, biomaterials are widely used to cover or fill the defects in the human body and organs. Biomaterials-based wound dressings are available but most of them are not cost-effective. They also rarely provide added advantages such as anti-bacterial properties and fast healing with improved angiogenesis. New biomaterials-based wound dressing should have the ability to deliver cytokines and growth factors at the wound site. Some efforts have already been made to develop biomaterials-based scaffolds with incorporated growth factors. The loaded growth factors are used to induce the expression of endogenous growth factors [8,9].

Proliferation, one of the key phases in healing, is mediated by a number of growth factors including Vascular Endothelial Growth Factor (VEGF) [10]. It is inhibited in the first step of wound healing, hemostasis, to promote clot formation. Then, the process of angiogenesis goes into the inflammatory phase, where the migration of inflammatory cells is directed to the wound site [11]. Multiple strategies have been developed in order to enhance acute and chronic wound healing, and among them, glycosaminoglycans are considered one of the good options [12].

Alginate is a biopolymer, which has been widely used as a wound dressing due to its easy availability, biocompatibility and biodegradability. It is also non-toxic and has a high absorption capacity [13]. Heparin, a member of the polyanionic polysaccharide family, is another type of glycosaminoglycans present in the extracellular matrix of blood vessels [14]. It is commonly used as an anticoagulant and has been suggested to be used in wound healing [15,16] due to its binding sites that bind to key growth factors and can help vascular cell attachment and migration [17]. It has anti-inflammatory properties which help in the process of wound healing, especially wounds caused by burns. It binds to many growth factors such as Transforming Growth Factor (TGF), VEGF and Fibroblast Growth Factor (FGF) [14,18]. It is widely used due to its anti-inflammatory and other pharmacological properties [19]. It binds to a wide number of proteins and helps in regulating their function [20]. Thus, it activates many growth factors and helps in promoting the process of angiogenesis. It has also been reported to ease pain and inflammation and reinstates the blood flow in burn patients [21,22]. Clinical trial data have shown that the application of heparin on skin with chronic wounds resulted in quick and enhanced wound healing [23]. It is found to be effective when applied topically, as it promotes tissue repair in rabbit trachea [24]. The property of heparin binding to its growth factor makes it a potential candidate for several therapeutic purposes [25,26]. Heparin was incorporated in alginate and arginyl-glycyl-aspartic acid (RGD) hydrogel for the purpose of increasing angiogenesis [14]. In another study, hydroxyapatite-integrated heparin- and glycerol-functionalized chitosan-based hydrogels were synthesized and were found to be effective in tissue regeneration [27]. The aim of this study was to prepare heparin-loaded alginate biomaterials by loading heparin drug on commercially available alginate wound dressings, which has not been previously studied considering its significance in stimulating wound healing mechanisms. The results showed that there was an increase in the angiogenic and antimicrobial potential of already present alginate wound dressing with the addition of heparin. Possible molecular mechanisms through which it promotes angiogenesis and healing were also evaluated.

## 2. Materials and Methods

### 2.1. Materials

Heparin (Heparin Inj. 5000IU, Leo) was obtained and stored at 4 °C. Commercially available Alginate wound dressing was supplied from Activheal^®^. Dulbecco’s Modified Eagle Medium (DMEM), Fetal Bovine Serum (FBS), Pen-Strep and trypsin were supplied from Gibco (Thermo Fisher Scientific, Waltham, MA, USA).

### 2.2. Loading of Heparin on Alginate Wound Dressings

Heparin was loaded onto the commercially available alginate dressings under sterile conditions. For this, three different concentrations of heparin (1 µg/mL, 5 µg/mL and 10 µg/mL) were used according to our previous report [28]. Briefly, 60 mm patches of pre-sterilized alginate dressings were cut, and syringe-filtered solutions of heparin with different concentrations were loaded onto alginate patches in biosafety cabinet and dried at room temperature under sterile environment. The final products were heparin-loaded alginate and labeled as A (1 µg/mL), B (5 µg/mL) and C (10 µg/mL).

### 2.3. FTIR (Fourier Transform Infrared Spectroscopy)

To demonstrate the chemical structure of heparin and heparin-loaded alginate wound dressing FTIR spectra were obtained using a spectrometer (Nicolet 6700 FT-IR spectrometer, Thermo Scientific, USA). Spectra were recorded at room temperature within 4000–500 cm^−1^ range under nitrogen environment and with carbon background using smart ATR mode.

### 2.4. Swelling Studies

Gravimetric technique was conducted to check the absorption uptake of the hydrogels and absorbance of exudates during wound healing. Swelling studies were carried out by soaking hydrogels in 1× PBS (pH 7.4) at 37 °C. Swollen hydrogels were measured after predetermined time. The experiment was done in triplicates. The dynamic weight change of the hydrogels with respect to time was calculated according to the formula:

S = [(W_S_ − W_D_)/W_D_] × 100

where W_S_ represents weight of swollen sample and W_D_ represents weight of dry sample [29].

### 2.5. Antimicrobial Assay

Antibacterial activity of heparin-loaded alginate samples was conducted using disc diffusion method [30], with slight modifications. For this assay, two bacterial strains: gram-negative *Escherichia coli* and gram-positive *Staphylococcus aureus* were used. The absorbance of both culture broths was set at 0.1 OD [31] and they were uniformly spread on the nutrient agar petri plates which were then incubated for 24 h at 37 °C. Antibacterial activity of hydrogels was determined by measuring zones of inhibition around them as compared to only alginate–hydrogel (control).

### 2.6. Cell Culture

NIH3T3 cells (fibroblasts) were thawed at 37 °C in water bath. Cells were cultured in plastic tissue culture flasks under sterile conditions, containing 4 mL Dulbecco’s Modified Eagle’s Medium (DMEM) supplemented with Fetal Bovine Serum (FBS) (10%), and penicillin-streptomycin (1%). After 80% confluency, cells were subcultured using Trypsin-EDTA and incubated in a humidified atmosphere at 37 °C with 5% CO_2_. Media was changed every 2 to 3 days. After 70% confluency, NIH3T3 cells were exposed to hydrogels (Control, A, B and C) and incubated at 37 °C, with 5% CO_2_, for days 1, 5 and 7. Cell viability and growth were observed under microscope (OPTIKA^®^, Ponteranica, Italy) [32].

### 2.7. In-Vitro Biocompatibility Assay

In-vitro biocompatibility assay was done to check the compatibility of fibroblasts NIH3T3 cells with the biomaterials. Alginate and heparin-coated alginate discs were exposed to cells. These well plates were then incubated at 35 °C with 5% CO_2_ for 24 h. After 24 h, attachment of cells was observed with samples. The experiment was done in triplicates.

### 2.8. Cell Viability Assay

To evaluate the effect of hydrogels on NIH3T3 cell line, Alamar blue assay was performed. After culturing cells in well plates, they were exposed to hydrogel extracts (samples soaked in cell medium) with dilution of 1:1 with culture media. Well plate was then incubated at 35 °C with 5% CO_2_. Working Alamar blue solution (100 μL) was added to each well and optical density at 570 nm, was recorded after incubation of 4 h by microplate spectrophotometer (Multiskan SkyHigh Microplate Spectrophotometer, Thermo Fisher Scientific, USA). The analysis was performed in triplicate. Control value was set at 100% viable and all values were determined as a percentage of the control. The values were analyzed by GraphPad prism 8.0 software (GraphPad Software, Inc., San Diego, CA, USA) and the percent viability of cells in three independent experiments is presented as mean ± SD [33].

### 2.9. RNA Extraction and Quantitative RT-PCR

Hydrogels’ treated cells lysate was used for RNA isolation by using a commercially available kit (Gene JET RNA Purification) according to the manufacturer protocol (Thermo Scientific, # K0731, USA). RNA was extracted in triplicates [34]. For reverse transcription, cDNA was synthesized by using a commercially available kit (RevertAid™ H Minus First Strand) by following the manufacturer’s instructions (Thermo Scientific, # K1622, USA). Amplification was done by real-time PCR (Applied Biosystems 7900HT Fast Real-Time PCR System) using kit (Maxima SYBR Green/ROX qPCR Master Mix (2X), Thermo Scientific, USA), following manufacturer’s guidelines (Thermo Scientific, # K0221, USA). The relative expression levels of each target gene were calculated using the Ct method by using Beta-actin (β-actin), and Relative mRNA expressions were analyzed using the ΔΔCt equation.

### 2.10. Wound Scratch Assay

The proliferation and the migration capability of NIH3T3 fibroblasts after exposure to our hydrogels were observed by doing a time-dependent wound healing scratch assay which measures the growth of cell population on the well plates surface. Cells were seeded onto a 6-well tissue culture plate. After certain confluency, samples were placed, and linear wound was generated. Three representative images from three wells of the same sample were taken at 0 h and 24 h to estimate the mean migration rate of cells [35]. The data were analyzed using (Graph Pad prism 8 software). The experiment was performed in triplicate [36].

### 2.11. Statistical Analysis

All the experiments were performed in triplicates and expressed as mean ± S.D. To measure the statistical difference, Two-way Anova was performed by using Graph-Pad Prism 8 software. *p*-value less than 0.05 was considered statistically significant (* *p* < 0.05, ** *p* < 0.01, *** *p* < 0.001 and **** *p* < 0.0001).

## 3. Results

### 3.1. FTIR Analysis: OH Groups and the Homogeneity of Both Heparin and Alginate

**Figure 1:**
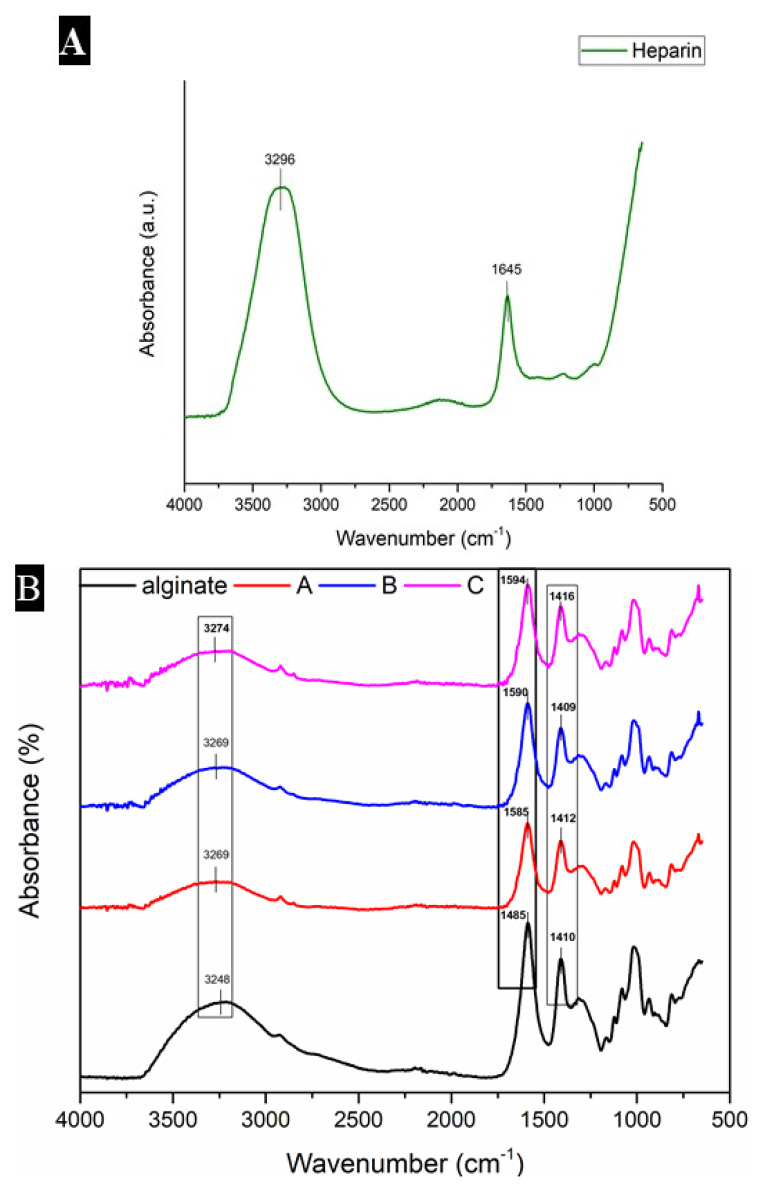
This figure shows the FTIR analysis of heparin (**A**), and other samples (**B**). In Figure 1B, the spectra of “alginate” represent control, “A” represents sample A, “B” represents sample B and “C” represents sample C in the spectral region (4000–500 cm^−1^).

The peaks in spectra of alginate and heparin-loaded alginate samples were analyzed by FTIR. The characteristic peaks of alginate in Figure 1 were observed at 1406 cm^−1^ and 1585 cm^−1^ [37], and the characteristic peaks of heparin were observed at 3296 cm^−1^ and 1645 cm^−1^. The peaks observed at 3218 cm^−1^, 3294 cm^−1^, 3296 cm^−1^, 3270 cm^−1^ and 3274 cm^−1^ indicated that all the samples have OH groups that come from both alginate and heparin. All the peaks were quite similar, which indicated the homogeneity of both heparin and alginate [38].

### 3.2. Swelling Studies Showed Increased Absorption by Heparin

**Figure 2:**
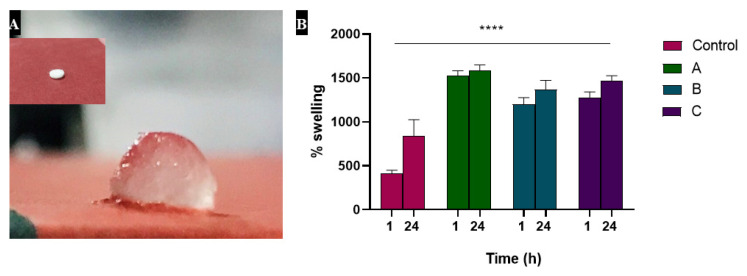
Shows swelling behaviors of hydrogels, where (**A**) represents the physical appearance of hydrogels before and after swelling while (**B**) represents the % degree swelling of hydrogels in PBS solution at 1 h and 24 h.

After soaking in PBS, swelling of the samples was observed by their weight. Most of the absorption occurred during 1 h, although the weight was slightly increased when checked after 24 h. This indicates the capability of hydrogels to absorb exudate and heparin-loaded alginate samples showed greater absorption (1524% swelling of A, 1202% swelling of B and 1279% swelling of C) relative to the control (alginate) sample which was swelled up to 838% as shown in Figure 2.

### 3.3. Heparin Increased the Antimicrobial Potential of Alginate Dressing

**Figure 3:**
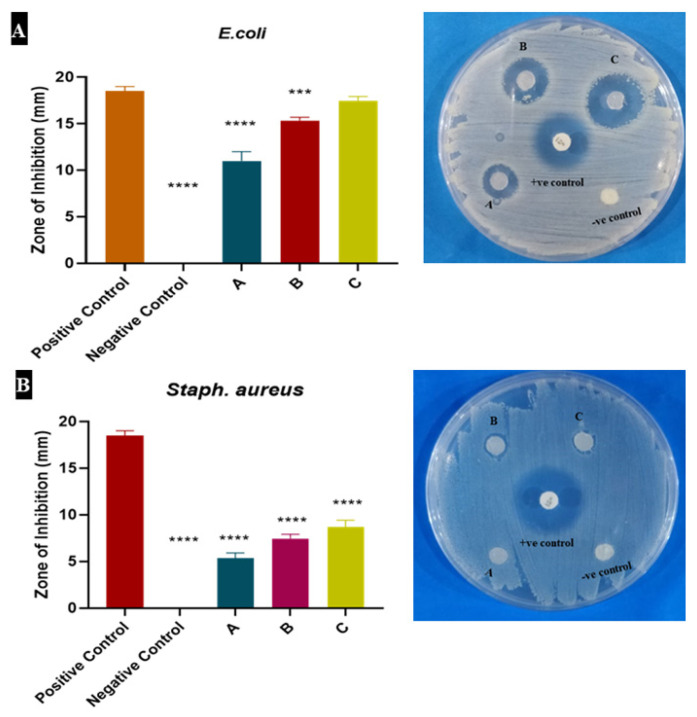
Antibacterial activity of alginate and heparin loaded alginate hydrogels by agar diffusion method: Zones of inhibition of hydrogels against *E. coli* (**A**) and *Staph. aureus* (**B**).

Control (Alginate) and heparin-loaded alginate (A, B and C) samples were tested for antibacterial assay by agar diffusion method. After 24 h incubation, the antibacterial activity of samples and control (both negative and positive) was observed by the zones of inhibition (ZOI) which showed the reduction in the growth of bacteria within the zones. These zones were also measured. Heparin-loaded alginate samples showed good antibacterial activity against *E. coli* and *Staph. aureus*, while alginate did not indicate any antibacterial activity. Among all samples, one having a 10 µg/mL (C) concentration of heparin showed the best antibacterial activity again *E. coli*. Samples as shown in Figure 3, inhibited gram-negative *E. coli* more than gram-positive *Staph. aureus*.

### 3.4. Heparin Loaded Alginate Dressings Were Highly Biocompatible

**Figure 4:**
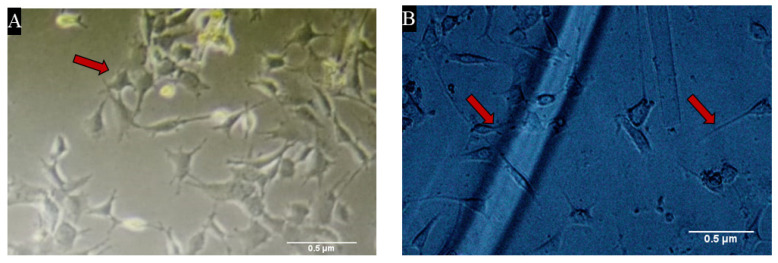
Shows biocompatibility of our biomaterials, where (**A**) shows the microscopic image of growing cells (indicated by the red arrows) in media with biomaterial while (**B**) represents the attachment of those cells with the fibers of samples.

An in-vitro biocompatibility test was done to check the cytocompatibility of samples. After 24 h, it was observed under the microscope that all the samples were biocompatible. Cells were seen to be attaching and proliferating with the sample fibers in 6 well plates as shown in Figure 4.

### 3.5. Heparin-Loaded Hydrogels Did Not Show Any Cytotoxic Effect on Cells

**Figure 5:**
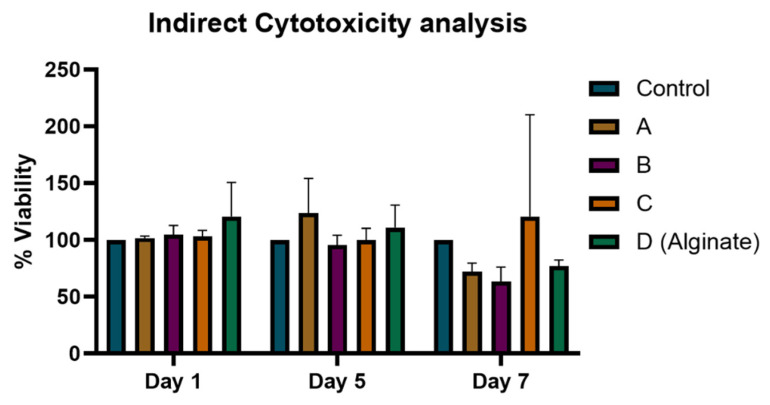
Shows cell viability of the heparin-loaded alginate dressings by Alamar Blue assay using Fibroblasts (NIH-3T3) cells with samples A, B and C vs. Controls at 570 nm wavelengths on days 1, 5 and 7 respectively.

To quantify the biocompatibility of samples, the Alamar Blue assay was done by using Alamar blue reagent to check the cell viability and proliferation in the presence of sample extract. The percentage viability values are given in Figure 5 in which control shows only cells. The viability of cells was increased to maximum in presence of 5 µg/mL of heparin on day 5. The proliferation of cells was slightly changed and increased to 120–150% on days 5 and 7, respectively, as compared to control with the passage of time, depending on the number of days they were incubated.

### 3.6. Hydrogels Induce VEGF and cPGE Synthase Expression

**Figure 6:**
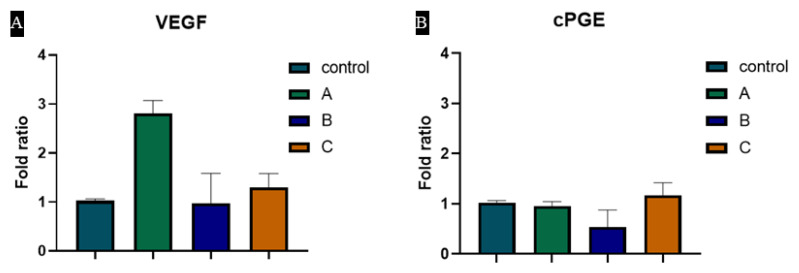
Expression of (**A**) VEGF and (**B**) cPGE synthase in different heparin (1 µg/mL, 5 µg/mL and 10 µg/mL) loaded hydrogels.

Regulation of VEGF and cPGE synthase were evaluated after treating with our biomaterial, i.e., alginate and heparin-loaded alginate dressings. Our results showed that VEGF is upregulated by “Sample A”, which had 1 µg/mL heparin. cPGE synthase, which maintains angiogenesis hemostasis and cellular growth [39], was also modulated by the heparin-loaded biomaterial. We presume that heparin-loaded alginate hydrogels may regulate the angiogenic process through these genes (Figure 6).

### 3.7. TNF-α and IL-1β Expression Was Downregulated by Heparin Coated Hydrogel

**Figure 7:**
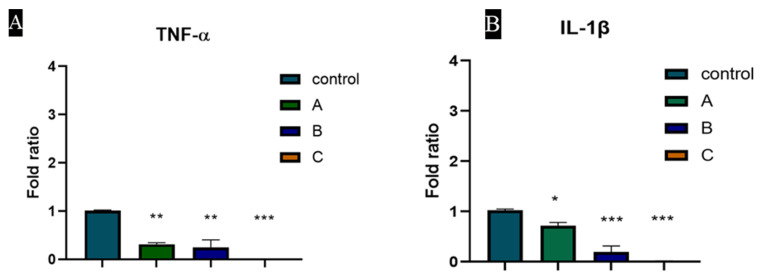
The expression of (**A**) TNF-α and (**B**) IL-1β in Fibroblasts, treated with different concentrations of heparin (1 µg/mL, 5 µg/mL and 10 µg/mL).

Expression of TNF-α and IL-1β were evaluated as shown in Figure 7. Heparin downregulates the expression of TNF-α which is the proinflammatory cytokine [40]. IL-1β, another potent proinflammatory cytokine was also reduced by heparin-containing biomaterials. The expression of TNF-α and IL-1β reduced in a concentration-dependent manner.

### 3.8. Cells Were Proliferated and Covered the Scratch in the Presence of Hydrogels

**Figure 8:**
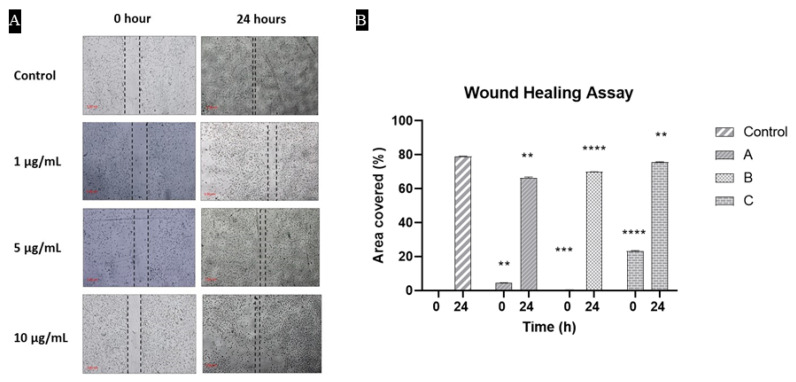
The migration rate of NIH3T3 cells determined by a scratch wound assay where (**A**) represents the microscopic images of migrating cells in different groups while (**B**) is the bar graph representing the migration rate in each group. Each bar indicates the mean of area covered when compared with the control.

Cells migration assay demonstrated the coverage of wound after 24 h. All generated wounds were almost covered regardless of the presence of the hydrogels. This indicated that cells were not disturbed morphologically and also the capability of hydrogel to promote the healing process by encouraging the proliferation of cells in the damaged area. In Figure 8, the extent of wound healing was quantified and presented in a bar graph.

## 4. Discussion

Wounds, damaged tissue or skin, can occur in any part of the body, either inside the body or outside. They are painful most of the time and cause discomfort to patients, which makes it difficult for patients to perform daily activities. Wound management is essential for better healing, which includes how to look after them while avoiding complications that can delay the healing process. Most traditional and cost-effective wound dressings only protect the wound from the external environment and do not accelerate the process of wound healing. Although many wound dressings are available in the market that enhance the process of wound healing along with some antimicrobial properties, they are expensive, and most people cannot afford them [41].

A previous study from our group [41] and other reports [42,43,44,45] have demonstrated the pro-angiogenic potential of heparin. Heparin, in combination with other biomaterials, increases angiogenesis (new vessel formation) and thus plays a role in the wound healing process. In our previous study, we also demonstrated the involvement of the VEGF gene in biomaterial-induced angiogenesis [46]. Keeping in view the pro-angiogenic and anti-microbial potential of heparin along with many other known therapeutic properties, we investigated the heparin-loaded alginate dressings. We wanted to characterize its properties and explore its compatibility with alginate, its overall bio-compatibility with cells, and molecular mechanisms for its angiogenic potential.

After preparing the hydrogels, different experiments including swelling studies, antimicrobial assay, cell culture assay and characterization were conducted. Characterization technique Fourier-transform infrared spectroscopy (FTIR) was used to check the peaks and bonds of heparin with alginate. Both heparin and alginate were found to be compatible with each other. Antimicrobial experiments revealed good antimicrobial activity of heparin against two pathogenic strains of two major groups of bacteria, i.e., *Staph. aureus* (gram positive) and *E. coli* (gram negative).

The absorbing ability of all the samples was checked, as they are known to be hydrophilic polymers that have the ability to become slow water-releasing hydrogels. Our results showed that heparin has the ability to swell and thus may play a role in absorbing exudates, which is one of the key factors in determining wound healing time [47,48,49]. According to our results, heparin did not induce any significant cytotoxic effect, which is in agreement with already reported studies [50,51]. Morphologically, cells looked healthy and viable, except for small and insignificant changes as compared to control, which is normal when cells are kept for longer.

Heparin plays an important role in a number of biological processes due to its ability to interact with growth factors and other biological substances. Sulfate and carboxylate groups leave heparin with a high negative charge, which electrostatically enables heparin to interact with many proteins, proteases and growth factors [52]. This makes it a good candidate to modulate angiogenesis in wound healing because such interactions in many cases give stability and increase the affinity for cell receptors [53]. Given its functions in angiogenesis, heparin may induce it through VEGF. That is why we included checking the expression of VEGF and other related genes in fibroblasts, treated with heparin. Our results demonstrated that VEGF, a proangiogenic growth factor, was upregulated in the presence of heparin-loaded alginate, which is in agreement with a previous report by Zieris et al., in 2011 [43]. According to our results, Sample A showed the maximum upregulation of VEGF and downregulated TNF-α which is a proinflammatory cytokine. Heparin tends to promote the interaction between cells and growth factors including VEGF, which in turn promotes the healing process by increased angiogenesis. Heparin also showed good anti-microbial activity against two strains of bacteria. There was a marked difference in the inhibition zones of heparin and control. All samples having different concentrations (1 µg/mL, 5 µg/mL and 10 µg/mL) of heparin showed good results in comparison to alginate sample (control). Sample A showed good absorption capability. It was also effective in alamar blue assay which gave minimum cytotoxicity and maximum proliferation of cells at day 5, while sample C was good in terms of antibacterial analysis.

## 5. Conclusions

In conclusion: our results revealed that heparin-loaded hydrogels have good absorption capability. When these hydrogels were exposed to NIH3T3 fibroblasts, they showed good biocompatibility and cell viability as their growth increased to around 140% on day 7. These wound dressings are potential stimulators of angiogenesis as well as able to prevent inflammation and infections at the wound site. Angiogenesis is regulated by VEGF and cPGE synthase involving various other downstream regulating genes and molecular pathways.

## Data Availability

All the relevant data are within the manuscript.
